# Tracing the Potential Flow of Consumer Data: A Network Analysis of Prominent Health and Fitness Apps

**DOI:** 10.2196/jmir.7347

**Published:** 2017-06-28

**Authors:** Quinn Grundy, Fabian P Held, Lisa A Bero

**Affiliations:** ^1^ Charles Perkins Centre Faculty of Pharmacy The University of Sydney Sydney Australia; ^2^ Charles Perkins Centre Faculty of Science The University of Sydney Sydney Australia

**Keywords:** mobile health, smartphone, privacy

## Abstract

**Background:**

A great deal of consumer data, collected actively through consumer reporting or passively through sensors, is shared among apps. Developers increasingly allow their programs to communicate with other apps, sensors, and Web-based services, which are promoted as features to potential users. However, health apps also routinely pose risks related to information leaks, information manipulation, and loss of information. There has been less investigation into the kinds of user data that developers are likely to collect, and who might have access to it.

**Objective:**

We sought to describe how consumer data generated from mobile health apps might be distributed and reused. We also aimed to outline risks to individual privacy and security presented by this potential for aggregating and combining user data across apps.

**Methods:**

We purposively sampled prominent health and fitness apps available in the United States, Canada, and Australia Google Play and iTunes app stores in November 2015. Two independent coders extracted data from app promotional materials on app and developer characteristics, and the developer-reported collection and sharing of user data. We conducted a descriptive analysis of app, developer, and user data collection characteristics. Using structural equivalence analysis, we conducted a network analysis of sampled apps’ self-reported sharing of user-generated data.

**Results:**

We included 297 unique apps published by 231 individual developers, which requested 58 different permissions (mean 7.95, SD 6.57). We grouped apps into 222 app families on the basis of shared ownership. Analysis of self-reported data sharing revealed a network of 359 app family nodes, with one connected central component of 210 app families (58.5%). Most (143/222, 64.4%) of the sampled app families did not report sharing any data and were therefore isolated from each other and from the core network. Fifteen app families assumed more central network positions as gatekeepers on the shortest paths that data would have to travel between other app families.

**Conclusions:**

This cross-sectional analysis highlights the possibilities for user data collection and potential paths that data is able to travel among a sample of prominent health and fitness apps. While individual apps may not collect personally identifiable information, app families and the partners with which they share data may be able to aggregate consumer data, thus achieving a much more comprehensive picture of the individual consumer. The organizations behind the centrally connected app families represent diverse industries, including apparel manufacturers and social media platforms that are not traditionally involved in health or fitness. This analysis highlights the potential for anticipated and voluntary but also possibly unanticipated and involuntary sharing of user data, validating privacy and security concerns in mobile health.

## Introduction

Mobile health is an exploding market, having doubled in just 2.5 years and reaching over 100,000 apps with market revenues projected to grow to US $26bn by 2017[1]. Every major technology company, including Apple, Google, and even Uber, has signaled an intention to enter the “digital health” market [2]. In mobile health, diverse stakeholders including companies, health professionals, and consumers perceive the collection, analysis, and sharing of user data to be of especially high value. The promise of big health data heralds shifts in health care toward consumer self-management through wearables and mobile apps with a focus on prevention and optimization [3]. For consumers, health and fitness apps also promise crowd-sourced information and social networks of support [4]. Researchers are also designing and testing methods to generate insights from the wealth of information shared over social media, such as Twitter, by users of mobile health and fitness apps [5].

A great deal of consumer data, collected actively through consumer reporting or passively through sensors, is shared among apps. App developers increasingly allow their programs to communicate with other apps, sensors, and Web-based services, which are promoted as features to potential users. This is typically done through shared application programming interfaces (API). A survey of 5000 app developers, representing almost 11,000 mobile health apps found that fitness and nutrition apps are among the most advanced in sharing via APIs [6]. This sharing most commonly occurs with data aggregators (eg, Apple’s HealthKit, a software platform that pools data from multiple health apps), sensors such as wearables, and directly between apps [6]. However, app developers may also choose to share consumer data with a variety of tools including analytic tools, social media APIs, performance tools and digital advertisers [6].

Apps serve an increasingly broad range of functions, some of which involve access to and the creation of vast amounts of highly personal data including a user’s location, text messages, or access to the mobile phone’s camera or photos [7]. Much of this information may be essential to the app’s functionality; however, the widespread collection, retention, and sharing of user data through apps has also created concern related to consumer privacy and the security of health-related data [8-10]. Because health apps by design often have access to personal health information, they pose a higher risk to consumers’ privacy; this information is often highly valuable to third parties, heightening the risk [10]. However, health apps also routinely pose risks related to information leaks, information manipulation, and loss of information [10]. There has been less investigation into the kinds of user data developers are likely to collect, and who might have access to it [7]. For example, Li developed a privacy threat model based on a health-related social networking site that accounts for multiple actors in the data usage and sharing network [11]. One principle threat accounted for in this model is user profiling across multiple sites: third parties can link multiple user accounts across apps to create aggregated user profiles and a more complete picture of a consumer’s social network. These aggregate profiles are then monetized and used for marketing, screening prospective tenants or employees, or maliciously for identity fraud [11].

Generating insights from mobile health data that can be translated into public health benefit will largely depend on the actions of those who own these data and the decisions they make regarding what to collect, how it will be aggregated and analyzed, and whether to share. Thus, it is essential to identify key actors and make their relationships transparent. Unfortunately, there is little transparency around these practices. The objective of this study was to describe how consumer data generated from mobile health apps might be distributed and reused with the aim of identifying potential user privacy and security risks. We investigated the self-reported collection and sharing of user data, while recognizing that this likely underreports the extent of both data collection and distribution. We investigated the nature of user data collection in the form of “permissions” that developers requested. We also traced the network of self-reported data sharing among a sample of prominent mobile health and fitness apps available in the United States, Canada, and Australia to better understand the potential for user data distribution within these networks.

## Methods

### Study Design

We conducted a structured content analysis of a purposive sample of prominent health and fitness apps available in the United States, Canada, and Australia. We then conducted a social network analysis of apps’ self-reported data sharing possibilities to understand how consumer data collected through the mobile platform might travel through this network.

### Sampling

We generated a purposive sample of prominent apps available in the United States, Canada, or Australia Google Play and iTunes app stores during November 2015. We employed purposive sampling to identify apps that were most likely to have data sharing ties, meaning they were likely to impact a large number of consumers. Due to the localized and personalized nature of app store search algorithms and rapidly changing population of apps, we triangulated two sampling strategies [12]: (1) a crawling program that systematically sampled the top-ranked 100 apps from the iTunes and Google Play app stores in the United States, Canada, and Australia and (2) purposive sampling of high-profile apps. These strategies were complementary by allowing exploration of data sharing relationships for developers that are both “well established” or “up and coming” so as to capture the dynamic nature of app development.

We first systematically sampled apps on a weekly basis that were ranked by the app stores as “top 100” using a crawling program that interacted directly with the stores’ API and automatically extracted the apps’ metadata. We identified 441 apps that were ranked in the top 100 in at least one country store during November 2015.

Beginning on November 1, 2015, we screened mainstream media (BBC, The New York Times, and The Guardian) and industry newsletters (MobiHealth News and RockHealth weekly) on a daily basis. We extracted the metadata for any health-related app receiving coverage. We continued screening until we generated a sample of 50 apps, which we determined would complement our systematic sample, allowing some representation for apps new to the market. We reached a sample of 50 on January 21, 2016.

Two researchers independently screened the sample of 491 apps for inclusion and excluded obvious duplicates. The inclusion criteria were that the app: explicitly pertained to a medical (eg, diabetes) or health condition (eg, obesity), health risk factor (eg, smoking), or health behavior (eg, walking), and provided guidance or a recommendation (eg, a workout program), tracked or recorded personal data, or made a health claim (eg, “improve heart health,” “lose weight,” or “reduce your anxiety”). Discrepancies were resolved through discussion until consensus was reached with input from a third researcher when necessary.

### Data Collection

We created an a priori coding instrument, which has been published in REDCap [13], a secure, Web-based application used to collect and manage data hosted at The University of Sydney (Multimedia Appendix 1). The instrument, based on a systematic review of methods for app content analysis [12], covered 4 domains: (1) app characteristics, (2) partnerships and affiliations, (3) developer and funding characteristics, and (4) permissions. We piloted the instrument on a sample of 70 randomly selected apps from our sample to ensure comprehensiveness of survey items and a high degree of inter-rater reliability.

We extracted data between December 12, 2015 and April 1, 2016 from app store descriptions, websites linked from the app store description and Google searches, with Google Play as a default content source when apps were distributed in both app stores. Two researchers independently extracted data related to partnerships and affiliations, defined as “any mention of a branded product, service or company, especially noting partnerships, collaborations, sponsors (funders), or brands,” and resolved any discrepancies through discussion until consensus was reached.

For apps available in Google Play, developers disclose how their apps will interact with the user’s device and personal information through reporting permissions [7]; Apple does not require developers to report permissions for apps distributed through iTunes. Permissions data were extracted for apps from the Google Play store description as reported by developers. Google encourages developers to request the minimum number of permissions required for an app’s functionality [14]; we did not, however, judge whether this was the case.

### Data Analysis

We inductively categorized relationships within the category “partnerships and affiliations,” one of which was data sharing. We defined instances of data sharing as any mention of a digital app, website, platform, sensor, wearable, or other smart device that was reported by the developer as a “partner” or as having the ability to share data. Examples of these promotional-type messages included: "Integration with Google Fit & MyFitnessPal — your running apps [sic] perfect companion”; or, “Easy, automatic exercise calorie counter, syncing with Fitbit, Withings, Jawbone and Garmin trackers and weight scales.”

The spelling of names for a large number of apps varied between platforms, countries, and store descriptions. These included variations in spelling (“plus”, instead of “+”), free and paid versions of the same app, and different naming practices between iTunes and Google Play. We grouped different instances of the same app through a two-stage process, first identifying similar names of apps automatically using approximate string matching, with a second author cross checking results [15].

We conducted descriptive analyses of app and developer characteristics in Microsoft Excel. We conducted an analysis of the network of sharing of user data among apps to understand the potential for data distribution in R using analysis packages igraph (1.0.1) and tnet (3.0.14). For this analysis, we grouped sampled apps together into app “families” that were offered by the same developer or owned by the same entity, again using approximate string matching, with a second author cross checking and expanding groups by joint ownership [15]. This approach assumed that the app families shared a common owner who had access to the data that were collected by their family of apps and the power to formulate the apps’ terms and conditions that grant legal access and in most cases, ownership, of these data.

The apps in our sample reported sharing user data with other included apps as well as apps that were not originally sampled in the methods described above. Both the initially sampled and these secondary apps were included as nodes in the network analysis. We constructed the network by connecting these app “families” with data sharing links whenever data sharing was reported. As our data does not give us any insights into the content of shared data or the direction of the flow, we considered these ties to be undirected and binary.

Descriptive measures of social network analysis provided a summary of the observed network. We calculated network density as the ratio of observed versus theoretically possible connections in a fully connected network and degree as the number of neighboring nodes per app.

**Figure 1:**
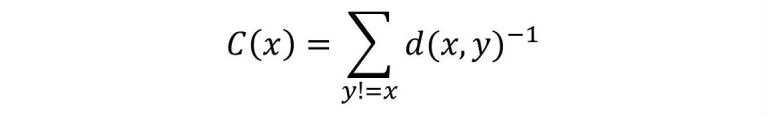
Closeness centrality index formula.

We quantified the position of an app (x) within the network by calculating its closeness centrality index. Closeness measures the potential for an app to access a piece of user data from anywhere in the network based on its network connections. Closeness to a single app (y) is defined as the inverse of the shortest path to y, or 0 if y cannot be reached from x. To calculate an index that compares all apps’ network positions, we summed up these values across all other apps to describe the position [16] (Figure 1).

Finally, random walk community detection was used to further specify how groups of apps relate to each other based on the structure of the network. This method identifies clusters of apps that are more closely connected either directly or through their neighbors [17]. This is achieved by simulating a process of random communication between neighboring apps, where a “message” is passed on from neighbor to neighbor for a certain number of steps. Essentially this process measures the probability that a message sent from x ends up at y.

## Results

The sample included 297 unique apps published by 231 unique developers. The majority had been sampled using the crawling program 265 (265/297, 89.2%) and 32 (32/297, 10.8%) from media sources. The majority of apps were available in both Google Play and iTunes (202/297, 68.0%), were free to download (172/297, 57.9%) and provided a link to a privacy policy in store or on the linked website (217/297, 73.1%).

### User Data Collection

We obtained data on permissions requested for the 241 apps that were available in Google Play, as iTunes does not report this data. Apps requested a total of 58 different permissions (mean 7.95, SD 6.57), ranging from 0 to 32 different permissions requested per app. The most common types of permissions requested related to Internet access including “full network access” (228/241, 94.6%) and “view network connections” (218/241, 90.5%). Seven apps did not request any permission. Two apps each requested 32 different permissions: Huawei Wear and Under Armour Record. All of the 26 apps that requested 20 or more different permissions were inductively categorized as activity monitors, which make use of GPS or an accelerometer such as UP by Jawbone, Map My Ride, or Nike+ Training Club, or as multi-focus health apps, which provided tailored insights related to diet, physical activity and sleep, such as Noom Coach and Microsoft Health.

Google Play classifies app permissions as “normal” or “dangerous” [14]. A “normal” permission requires access to data or resources outside of the app, but there is little risk to a user’s privacy or the operation of other apps; for example, control over the phone’s vibration. “Dangerous” permissions request data or resources that involve the user’s private information, stored data, or affect the operation of other apps; for example, granting the app access to a user’s contacts, confidential calendar information, or unique device ID. Of the 58 permissions requested by sampled apps, 26 are classified as “dangerous.” The most commonly requested “dangerous” permissions were “read storage” (195/241, 80.9% of sampled apps) and “modify storage” (189/241, 78.4% of sampled apps), which allow the app to read and write to the device’s internal storage.

### User Data Sharing

We grouped the 297 apps into 222 app “families” (ie, those which shared a developer or an acquiring company). These app families reported sharing data with 137 secondary app families, leading to a network of 359 app-family-nodes, with one connected central component of 210 app families (210/359, 58.5%). This meant that there were undirected paths of data sharing that connected 210 app families directly or indirectly. Of these 210 connected app families, 75 app families (75/210, 35.7%) were health app families in our initial sample. Most (143/222, 64.4%) of the sampled app families did not report sharing any data and were therefore isolated from each other and from the core network. Thirty app families (30/222, 13.5%) from our sample reported only a single tie to another app family; 119 secondary app families were only identified in a data sharing relationship once. Within the connected component, any 2 app families were connected by a maximum of 6 steps, and with an average of 3.28 connections between any two apps. The density of the entire network was 0.005, meaning that only very few of the theoretically possible connections between all the app families were in fact realized.

**Figure 2:**
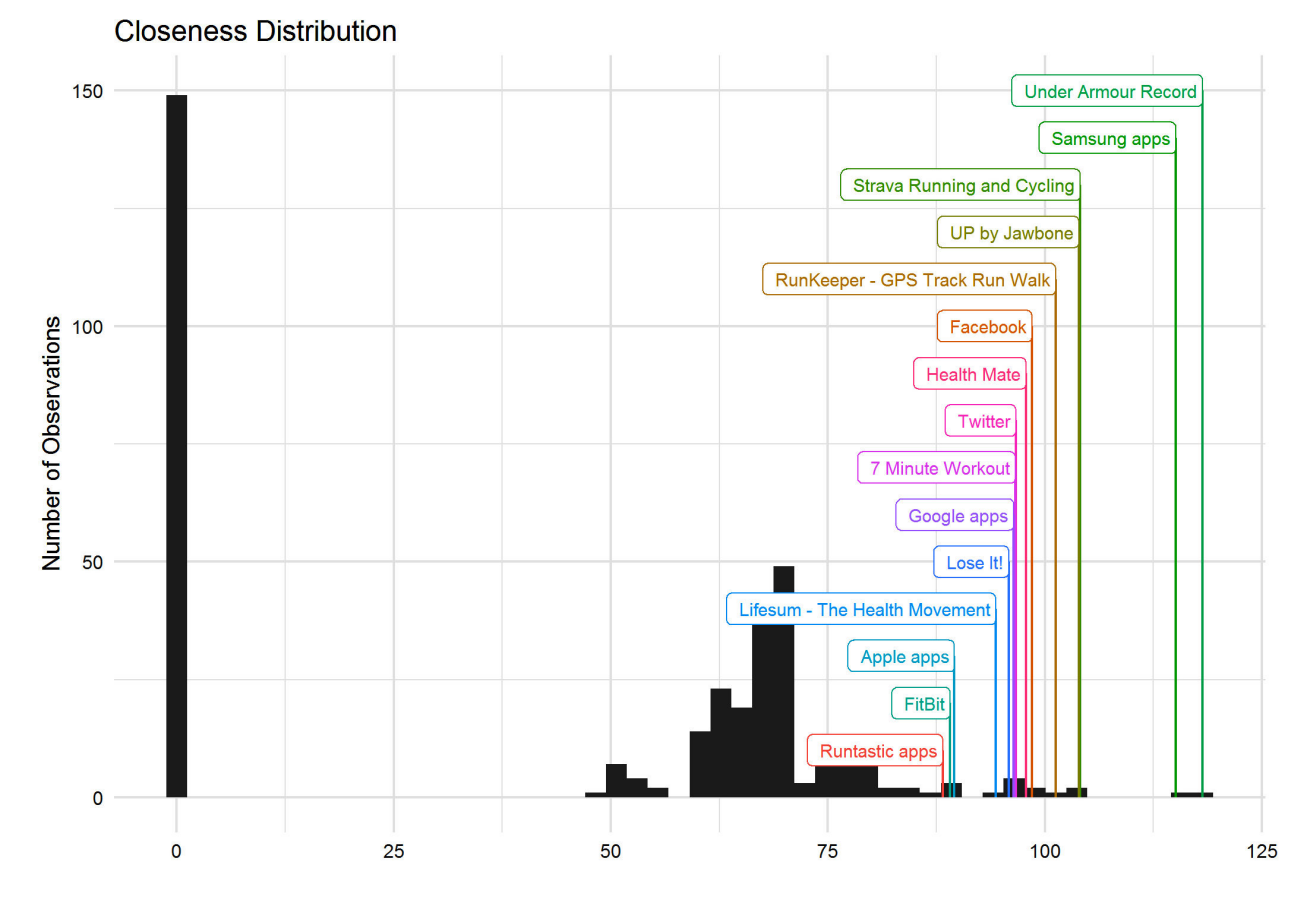
Distribution of the closeness centrality index highlighting the top fifteen entries.

The distribution of closeness centrality scores clearly separated the isolated app families and revealed a skewed distribution of app families’ positions in the core. We highlight the 15 highest scoring app families in Figure 2. These families take on particularly important roles in the network: they may be distributing or receiving user-generated data with many other apps. Table 1 describes characteristics of these fifteen central app families, 12 of which included apps that were in our original sample. The majority of these central app families were publicly traded corporations (10/15, 67%) and represented the technology (wearables, mobile health, and social media) and fashion (sports apparel) sectors.

**Table 1:** Characteristics of centrally-positioned app families.

App family	Developer	Developer type	Sector
7 Minute Workout	Wahoo Fitness	Privately held	Wearables
Under Armour apps (under Armour Record, MyFitnessPal, MapMyFitness, Endomondo)	Under Armour Inc	Publicly traded	Sports apparel
RunKeeper - GPS Track Run Walk	FitnessKeeper, Inc. (acquired by ASICS)	Publicly traded	Mobile health
Lose It!	FitNow, Inc.	Privately held	Mobile health
Google apps (eg, Google Tracks, Gmail, Google Drive, Google Maps, Google Wallet, Google+, YouTube)	Alphabet Inc.	Publicly traded	Technology
Health Mate	Withings (acquired by Nokia)	Publicly traded	Technology
UP by Jawbone apps	Jawbone	Privately held	Wearables
Lifesum - The Health Movement	Lifesum	“Start-up”	Mobile health
Samsung apps (eg, S Health, SmartThings)	Samsung Group	Publicly traded	Technology
Strava Running and Cycling	Strava	Privately held	Mobile health
Facebook	Facebook, Inc.	Publicly traded	Social media
Twitter	Twitter Inc.	Publicly traded	Social media
Apple apps (eg, HealthKit, iTunes, Apple Watch, Apple TV)	Apple Inc.	Publicly traded	Technology
Fitbit	Fitbit Inc	Publicly traded	Wearables
Runtastic apps (eg, Runtastic Running & Fitness, Runtastic Me, Runtastic Road Bike)	Runtastic (acquired by Adidas)	Publicly traded	Mobile health

Finally, we simulated a random communication process between these app families, using the data-sharing network as the channel of information transmission. This allowed us to identify groups of app families that may be more likely to share user data with each other, as they are more tightly connected with each other in the network. The resulting network is shown in an interactive visualization in the Multimedia Appendix 1. The size of a dot represents how it was sampled: app families in our initial sample are bigger. Colors indicate the membership to communities identified through the walktrap clustering algorithm. We chose to simulate a random communication process of 4 steps because more steps did not noticeably increase the results’ modularity. The result shows one large group of health and fitness app families that tended to link with each other (light blue). This group also included the major manufacturers of mobile devices and their respective health and fitness data aggregators (Google Tracks (now defunct)/Google Inc., HealthKit/Apple Inc. and S Health/Samsung Group). Furthermore, there were several communities that fanned out from individual apps. These included Samsung Apps, 7 Minute workout, and UP by Jawbone. Similar patterns were seen for apps that were not in our initial sample, including Apple apps, Facebook, and Twitter. In fact, the two social networking sites connected to the same community of app families.

## Discussion

### Overview

This analysis provides a cross-sectional view of the possibilities for user data collection and potential paths that these data are able to travel among a sample of prominent health and fitness apps. The implication for consumers is that while individual apps may not collect personally identifiable information, app families and the partners with which they share data, may be able to aggregate consumer data, achieving a much more comprehensive picture of the individual consumer. We used information that was self-reported by developers in promotional materials such as app store descriptions as a proxy for forms of technological communication such as shared APIs. Thus, it could be inferred that the connections between apps in this study are those that developers choose to advertise, which users may value. This also means that this analysis likely underrepresents the extent of sharing of user data, as it cannot be presumed that user-selected networks are the lone third parties accessing consumer data [18]. For example, the US Federal Trade Commission analyzed 12 health and fitness apps and found they shared data such as usernames, names, email addresses, postal codes, geo-location, and exercise and diet habits with 76 third parties [19]. The extent of user data collection and sharing described here may only be the tip of an iceberg.

### Principal Findings

Despite capturing only a fraction of the extent of user data sharing, we found that a core group of app families are sparsely connected and may be exchanging user data, though this analysis is not able to determine the directionality of exchange. This opens up new and important avenues for research: both into the ways that user-generated data is actually being shared among mobile health apps and third parties, but also and perhaps more importantly, into the ways that this data is combined and used.

The market of mobile health and fitness apps displays a remarkable dichotomy: most app families are stand-alone offerings that do not report any integration with the rest of the market. However within the connected core of app families, there are a number of hypothetical pathways for the sharing of users’ data between app families and third parties, such as social media sites. Fifteen app families held crucial positions that may enable them to act as gatekeepers in the flow of data and to gather user information from diverse sources. Notably, the organizations behind these app families represent diverse industries, including apparel manufacturers and social media platforms that are not traditionally involved in health or fitness. Three entities—Apple, Facebook, Inc. and Twitter Inc.—were not even present in our original sample, yet assumed central positions in the network. These central network positions may also amplify an entity’s ability to aggregate consumer-generated data, which has not only privacy, but market implications. These central network positions may be akin to monopolies given the value of consumer data, and anti-trust regulators are working to ensure data competition [20]. App developers are likely incentivized to build Facebook, Twitter or Apple Watch connectivity through their APIs due to their large consumer bases, which may put developers that are more circumspect about data sharing at a competitive disadvantage.

On an average, our sample of Android health and fitness apps requested a higher number of permissions than apps in general [7]. Pew Research Center analyzed over 1 million apps available in Google Play in 2014 and found that apps requested on average 5 permissions, in comparison to the nearly 8 permissions requested per health app in our sample [7]. Similar to apps in general, sampled Android health apps most commonly requested permissions relating to Internet access, which allows for the delivery of targeted advertising and the ability to access the phone’s storage, which allows apps to not only save content to the device, but also to access personal data stored by other apps [7]. Sampled Android health and fitness apps also tended to request “dangerous” permissions more frequently than apps in general [7].

These findings validate privacy and security concerns in mobile health due to the sensitivity of health-related data as well as the collection of more kinds of personally identifiable data [8,9]. Particularly, the third party use of personal health-related information could result in employment, housing, or education-related discrimination, loss of insurance coverage or higher premiums, predatory advertising, or medical identity theft [21-23]. However, the implications of a privacy violation or security breach related to user-generated data via mobile health apps are largely speculative, emergent or not well understood. For example, recent news coverage highlighted the value of even a mobile phone number as an unregulated and unique digital identifier that can be used to link a user’s personal data held by multiple companies from social networking sites to health apps to credit agencies [24]. This information is made available to developers and third parties when a user grants the permission “read phone status and identity.”

This analysis highlights the potential for anticipated and voluntary, but also possibly unanticipated and involuntary sharing of user data, though we could not distinguish between the two. Though users may willingly engage in sharing their data, such as posting to social media, they may not fully appreciate the nature of data collection or the extent to which sharing occurs. A study with Facebook users found that users were under-informed about the nature of data collection within the app, and that even after receiving explicit information, many still did not fully understand the extent to which apps could access personal data [25]. Though Google Play allows developers to disclose the permissions requested, these disclosures are not accompanied by non-technical, lay descriptions of a given permission, nor what the likely and possible implications of granting such permission entails. Similarly, the sharing and protection of user data is far from transparent. In a study of privacy policies of the 300 most frequently rated health apps in the Apple and Google Play app stores, researchers found only 30% had a privacy policy with an average college-level reading grade level, and 2 out of 3 did not specifically address the app itself [26]. Thus, as mobile app companies gain unprecedented knowledge about app users, consumers know very little or nothing about how this knowledge is used [22].

This network analysis similarly highlights the potential for the unanticipated travel of user data through networks of health and fitness apps and their supporting entities. In fact, regulators are beginning to take note of the way that user data travels within app networks. A German privacy regulator recently ordered Facebook to stop collecting and storing the data of WhatsApp users, a social messaging app it had acquired [27]. The regulators argued that Facebook infringed data protection law when it announced a policy change that would allow unprecedented use of WhatsApp users’ data, including phone numbers, without effective approval from users. Facebook is currently appealing this order [27].

### Limitations

Our analysis of a purposive sample of apps is based upon data collected over a 4-month period from December 2015 through April 2016. Due to the highly dynamic nature of the app marketplace, it is likely that some of the apps are no longer available and that data are out of date. As we relied upon developer self-report, we could not verify whether the permissions requested are actually being used by the sampled apps or how user data is then analyzed or shared, thus, they serve as a proxy for the type of transmitted information. As these permissions are self-reported and are only reported by Android developers, it is likely that this represents an underestimate of permissions requested. This analysis highlights the possibilities for the sharing of consumer-generated data, but it does not differentiate between the potential and actual flow of data or the direction in which data travels. For example, there may be practical barriers to data sharing or this exchange may be subject to user control. Future research should consider performing traffic analyses, such as a man-in-the-middle approach, to verify the nature and extent of consumer data sharing and protection [8]. Furthermore, our analysis does not distinguish between user data that is shared consciously by the consumer, such as posting a workout to social media, and the user data that is collected by the app passively through the permissions requested, such as reading a user’s contacts. However, this analysis does provide a unique snapshot in time that highlights the value of taking a network approach to studying health and fitness apps.

### Conclusions

The possibilities afforded by the collection of consumer-generated, health-related data through mobile health and fitness apps are vast. However, the ways that consumer-generated, and sometimes sensitive personal information, is shared and passed on within the mobile ecosystem is far from transparent. The connections among apps in this study are only those that developers choose to advertise, and thus are likely only the tip of the iceberg. Thus, concerns regarding privacy and security should apply to this mobile ecosystem and not apps alone. Several major players in mobile health and fitness hold key gatekeeping positions in the sharing of consumer data, which amplifies their ability to amass consumer-generated data. Policymakers should particularly account for these actors in ensuring consumer privacy, security, and market competition.

## Appendix

Multimedia Appendix 1 Network of reported data sharing, highlighting communities of densely connected apps that have a higher potential of sharing users’ data among each other, due to their direct and indirect connections. Bigger nodes were included in our initial sample, smaller nodes were mentioned by them as partners. Networks are presented using a force directed layout algorithm from the D3 library (http://d3js.org/).

## Abbreviations

API: application programming interface
